# Tris(ethane-1,2-diamine-κ^2^ *N*,*N*′)nickel(II) diiodide

**DOI:** 10.1107/S1600536809054580

**Published:** 2009-12-24

**Authors:** Greg Brewer, Ray J. Butcher, Jerry P. Jasinski

**Affiliations:** a Department of Chemistry, Catholic University, 620 Michigan Av. NE, Washington, DC 20059, USA; b Department of Chemistry, Howard University, 525 College Street NW, Washington, DC 20059, USA; c Department of Chemistry, Keene State College, 229 Main Street, Keene, NH 03435-2001, USA

## Abstract

The title compound, [Ni(C_2_H_8_N_2_)_3_]I_2_, crystallizes with an [Ni(en)_3_^2+^] cation (en is ethane-1,2-diamine) and two iodide ions in the asymmetric unit. Two of the en ligands surrrounding the Ni^2+^ ion have disordered C atoms, while the third exhibits extensive weak N—H⋯I inter­actions with the two iodide ions that extend throughout the crystalline lattice, producing an infinite network along (011).

## Related literature

For related structures, see: Cramer *et al.* (1976 ▶); Cramer & Huneke (1978 ▶); Korp *et al.* (1980 ▶); Raston *et al.* (1978 ▶); Swink & Atoji (1960 ▶); Wieczorrek (2000 ▶). For double salts, see: Alvarado *et al.* (2009 ▶); Brewer *et al.* (2007 ▶); Dvorkin *et al.* (1989 ▶, 1991 ▶); Farago *et al.* (1967 ▶). For a description of the Cambridge Structural Database, see: Allen (2002 ▶). For puckering parameters, see: Cremer & Pople (1975 ▶).
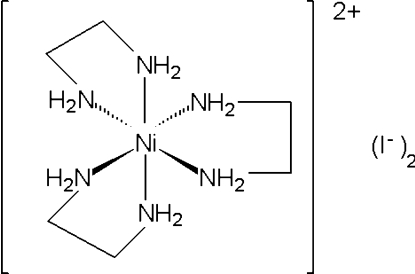


## Experimental

### 

#### Crystal data

[Ni(C_2_H_8_N_2_)_3_]I_2_
*M* *_r_* = 492.82
Orthorhombic, 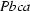

*a* = 14.7502 (6) Å
*b* = 13.4881 (4) Å
*c* = 15.9624 (7) Å
*V* = 3175.8 (2) Å^3^
*Z* = 8
Mo *K*α radiation
μ = 5.10 mm^−1^
*T* = 200 K
0.55 × 0.47 × 0.38 mm

#### Data collection

Oxford Diffraction Gemini R Mo diffractometer
Absorption correction: multi-scan *CrysAlis RED* (Oxford Diffraction, 2009 ▶) *T*_min_ = 0.337, *T*_max_ = 1.000
19070 measured reflections
5271 independent reflections
2835 reflections with *I* > 2σ(*I*)
*R*_int_ = 0.042

#### Refinement

*R*[*F* ^2^ > 2σ(*F* ^2^)] = 0.034
*wR*(*F* ^2^) = 0.091
*S* = 0.96
5271 reflections
161 parameters
36 restraints
H-atom parameters constrained
Δρ_max_ = 1.65 e Å^−3^
Δρ_min_ = −1.09 e Å^−3^

### 

Data collection: *CrysAlis CCD* (Oxford Diffraction, 2009 ▶); cell refinement: *CrysAlis RED* (Oxford Diffraction, 2009 ▶); data reduction: *CrysAlis RED*; program(s) used to solve structure: *SHELXS97* (Sheldrick, 2008 ▶); program(s) used to refine structure: *SHELXL97* (Sheldrick, 2008 ▶); molecular graphics: *SHELXTL* (Sheldrick, 2008 ▶); software used to prepare material for publication: *SHELXTL*.

## Supplementary Material

Crystal structure: contains datablocks global, I. DOI: 10.1107/S1600536809054580/pb2013sup1.cif

Structure factors: contains datablocks I. DOI: 10.1107/S1600536809054580/pb2013Isup2.hkl

Additional supplementary materials:  crystallographic information; 3D view; checkCIF report

## Figures and Tables

**Table:** 

Ni—N31	2.101 (3)
Ni—N22*A*	2.105 (7)
Ni—N12*A*	2.113 (7)
Ni—N21*A*	2.116 (7)
Ni—N32	2.122 (3)
Ni—N11*A*	2.140 (5)

**Table:** 

N31—Ni—N22*A*	93.25 (16)
N31—Ni—N12*A*	91.3 (3)
N22*A*—Ni—N12*A*	90.2 (2)
N31—Ni—N21*A*	171.6 (2)
N22*A*—Ni—N21*A*	81.1 (2)
N12*A*—Ni—N21*A*	94.9 (3)
N31—Ni—N32	81.99 (11)
N22*A*—Ni—N32	95.9 (2)
N12*A*—Ni—N32	171.2 (2)
N21*A*—Ni—N32	92.4 (2)
N31—Ni—N11*A*	97.93 (14)
N22*A*—Ni—N11*A*	166.02 (19)
N12*A*—Ni—N11*A*	81.26 (19)
N21*A*—Ni—N11*A*	88.6 (2)
N32—Ni—N11*A*	93.98 (14)

**Table 2:** Hydrogen-bond geometry (Å, °)

*D*—H⋯*A*	*D*—H	H⋯*A*	*D*⋯*A*	*D*—H⋯*A*
N31—H31*A*⋯I2	0.92	2.83	3.731 (3)	167
N31—H31*B*⋯I1	0.92	2.79	3.663 (3)	158
N32—H32*A*⋯I2^i^	0.92	3.05	3.786 (3)	138
N32—H32*B*⋯I1^ii^	0.92	2.86	3.724 (3)	157
N11*A*—H11*B*⋯I2^iii^	0.92	2.97	3.854 (4)	162
N12*A*—H12*C*⋯I2	0.92	3.15	3.940 (8)	145
N11*B*—H11*F*⋯I2^iii^	0.92	3.15	3.619 (12)	114
N12*B*—H12*G*⋯I2	0.92	3.07	3.94 (2)	159
N12*B*—H12*H*⋯I1^iv^	0.92	3.27	3.824 (16)	121
N21*A*—H21*A*⋯I2^i^	0.92	2.92	3.826 (8)	171
N21*A*—H21*B*⋯I1^i^	0.92	2.77	3.665 (7)	166
N22*A*—H22*D*⋯I1^ii^	0.92	3.19	3.905 (7)	136
N21*B*—H21*E*⋯I2^i^	0.92	3.05	3.86 (2)	148
N21*B*—H21*F*⋯I1^i^	0.92	3.22	3.832 (16)	125
N22*B*—H22*G*⋯I2	0.92	3.21	4.061 (16)	154
N22*B*—H22*H*⋯I1^ii^	0.92	2.80	3.69 (2)	163

Symmetry codes: (i) 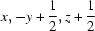; (ii) 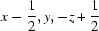; (iii) 
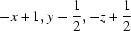
; (iv) 
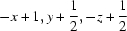
.

**Fig. 1.**
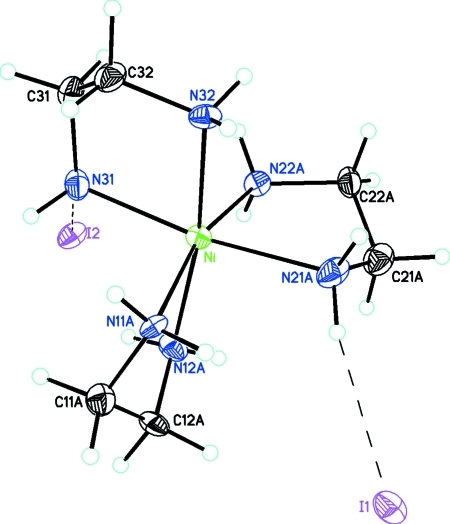
Molecular structure of the title compound, C6H24N6NiI2, showing the [Ni(en)32+] cation and two I_ ions in the asymmetric unit, the atom labeling scheme and 50% probability displacement ellipsoids. Only the major components of disordered carbon atoms (C11A, C12A & C21A, C22A) in rings 11 (Ni—N11—C11A—C12A—N12: 0.744 (12)) and 21 (Ni—N21—C21A—C22A—N22; 0.684 (9)) are depicted. Dashed lines indicate weak N—H···I hydrogen bond interactions.

**Fig. 2.**
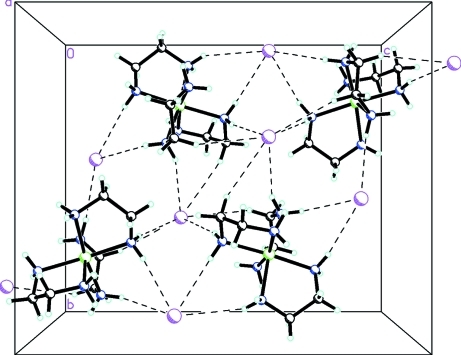
Packing diagram of the title compound, (I), viewed down the a axis. Dashed lines indicate weak intermolecular N—H···I interactions which produces a infinite weak bonding network arranged along the (011) plane of the unit cell.

## supplementary crystallographic information

### Comment

Recent work has shown that an iron(II) Schiff base complex of tris(2-aminoethyl)amine(tren) with imidazole-2-carboxaldehyde form double salts with metal (K^+^, Rb^+^, Cs^+^ and NH_4_^+^) perchlorates (Brewer *et al.*, 2007) and metal (*M* = Na^+^, K^+^, Rb^+^, Cs^+^ and NH_4_^+^) tetrafluoroborates (Alvarado *et al.*, 2009). Thus this system shows size selectivity for alkali metal cations. Structural studies of the thiocyanate salts reveal a linear polymeric anion, [(*M*(SCN)_3_)^2-^]_n_. [Ni(en)_3_]^2+^ and [Zn(en)_3_]^2+^ react with MX (*M* = K^+^ or NH_4_^+^, *X*=SCN^-^ or SeCN^-^–) to form double salts, [Ni(en)3](SCN)_2_.NH_4_(SCN) (Dvorkin *et al.*, 1991) and [Ni(en)_3_](SeCN)_2_.K(SeCN) (Farago *et al.*, 1967) or [Zn(en)_3_](SCN)_2_.K(SCN) (Dvorkin *et al.*, 1989). Many of these structures also reveal a linear polymeric anion, [(*MX*_3_)^2-^]_n_ (*X*=ClO_4_^-^ or BF_4_–), similar to that observed above suggesting that anions of this type are stable in certain settings and may be used in other reactions. The anions in all of the above complexes are tethered to the cations through hydogen bonds involving either amine, –NH_2_ or the bidentate hydrogen bonding donor, -*N*=C_imine_(*H*)—C_imidazole_—N_imidazole_(H). In an effort to determine the nature of the interactions between the amine, –NH_2_ (in the cation building unit used in the formation of double salts) and the anion in stabilizing these salts we report the crystal structure of the title compound, C_6_H_24_N_6_NiI_2_, (I).

C_6_H_24_N_6_NiI_2_, (I), crystallizes with a [Ni(en)_3_^2+^] cation and two I^_^ ions in the asymmetric unit (Fig. 1). In the cation, two of the 1,2-ethanediamine-N,*N*' rings surrrounding the Ni^2+^ ion contain disordered carbon and nitrogen atoms while the third exhibits extensive weak N—H···I interactions with the two iodide ions (Table 1) that extend throughout the crystalline lattice producing an infinite network along the (011) plane of the unit cell (Fig. 2). The major components of the two disordered 5-membered Ni^2+^-1,2-ethanediamine-N,*N*' rings adopt sightly distorted half-chair conformations (Cremer & Pople, 1975) with puckering parameters Q(2), and Phi(2) of 0.403 (2) Å, 86.767 (6)° (ring 11 = Ni/N11/C11A/C12A/N12; 0.744) and 0.423 (5) Å, 82.357 (0)° (ring 21 = Ni/N21/C21A/C22A/N22; 0.684), respectively. The Q(2), and Phi(2) values for ring 31 (Ni/N31/C31/C32/N32) are 0.438 (4)Å and 270.967 (6)°. For an ideal half-chair, Phi(2) = k *x* 36° + 18° or 180° + Phi(2). The dihedral angle between the mean planes of the normal ring (31) and the major components of the disordered rings (11 & 21) measures 85.6 (8)° and 83.5 (0)°, while between rings 11 and 21 themselves is 87.2 (7)°. Bond distances within the cation are normal (Allen *et al.*, 2002) and comparable to those in similar structures (Dvorkin *et al.*, 1989, 1991; Farago *et al.*, 1967; Cramer *et al.* 1976; Cramer & Huneke, 1978).

The presence of I1 and I2 in the crystal lattice allows for the formation of a collection of weak intermolecular N–H···I interactions which thereby influences crystal stability (Table 1).

### Experimental

Nickel(II) chloride hexahydrate was dissolved in water and excess ethylenediamine was added to the green solution. The resulting violet/purple solution was allowed to go to dryness. The crude [Ni(en)_3_]Cl_2_ was redissolved in water saturated with potassium iodide. The dark blockish crystals suitable for *x*-ray studies, in space group Pbca, were collected by filtration on standing.

### Refinement

All of the H atoms were placed in their calculated positions and then refined using the riding model with N—H = 0.93 Å, C—H = 0.95–0.99 Å, and with *U*_iso_(H) = 1.18–1.51*U*_eq_(C,*N*).

### Crystal data

**Table:**

[Ni(C_2_H_8_N_2_)_3_]I_2_	*F*(000) = 1888
*M**_r_* = 492.82	*D*_x_ = 2.061 Mg m^−^^3^
Orthorhombic, *P**b**c**a*	Mo *K*α radiation, λ = 0.71073 Å
Hall symbol: -P 2ac 2ab	Cell parameters from 8584 reflections
*a* = 14.7502 (6) Å	θ = 4.6–32.4°
*b* = 13.4881 (4) Å	µ = 5.10 mm^−^^1^
*c* = 15.9624 (7) Å	*T* = 200 K
*V* = 3175.8 (2) Å^3^	Prism, pale purple
*Z* = 8	0.55 × 0.47 × 0.38 mm

### Data collection

**Table:**

Oxford Diffraction Gemini R Mo diffractometer	5271 independent reflections
Radiation source: Enhance (Mo) X-ray Source	2835 reflections with *I* > 2σ(*I*)
graphite	*R*_int_ = 0.042
Detector resolution: 10.5081 pixels mm^-1^	θ_max_ = 32.5°, θ_min_ = 4.6°
ω scans	*h* = −21→19
Absorption correction: multi-scan *CrysAlis RED* (Oxford Diffraction, 2009)	*k* = −10→19
*T*_min_ = 0.337, *T*_max_ = 1.000	*l* = −24→16
19070 measured reflections	

### Refinement

**Table:**

Refinement on *F*^2^	Primary atom site location: structure-invariant direct methods
Least-squares matrix: full	Secondary atom site location: difference Fourier map
*R*[*F*^2^ > 2σ(*F*^2^)] = 0.034	Hydrogen site location: inferred from neighbouring sites
*wR*(*F*^2^) = 0.091	H-atom parameters constrained
*S* = 0.96	*w* = 1/[σ^2^(*F*_o_^2^) + (0.043*P*)^2^] where *P* = (*F*_o_^2^ + 2*F*_c_^2^)/3
5271 reflections	(Δ/σ)_max_ = 0.001
161 parameters	Δρ_max_ = 1.65 e Å^−^^3^
36 restraints	Δρ_min_ = −1.09 e Å^−^^3^

### Special details

**Table:**

Geometry. All e.s.d.'s (except the e.s.d. in the dihedral angle between two l.s. planes) are estimated using the full covariance matrix. The cell e.s.d.'s are taken into account individually in the estimation of e.s.d.'s in distances, angles and torsion angles; correlations between e.s.d.'s in cell parameters are only used when they are defined by crystal symmetry. An approximate (isotropic) treatment of cell e.s.d.'s is used for estimating e.s.d.'s involving l.s. planes.
Refinement. Refinement of *F*^2^ against ALL reflections. The weighted *R*-factor *wR* and goodness of fit *S* are based on *F*^2^, conventional *R*-factors *R* are based on *F*, with *F* set to zero for negative *F*^2^. The threshold expression of *F*^2^ > σ(*F*^2^) is used only for calculating *R*-factors(gt) *etc*. and is not relevant to the choice of reflections for refinement. *R*-factors based on *F*^2^ are statistically about twice as large as those based on *F*, and *R*- factors based on ALL data will be even larger.

### Fractional atomic coordinates and isotropic or equivalent isotropic displacement parameters (Å^2^)

**Table:**

	*x*	*y*	*z*	*U*_iso_*/*U*_eq_	Occ. (<1)
I1	0.611433 (17)	0.132498 (19)	0.122134 (16)	0.03800 (7)	
I2	0.362274 (19)	0.435966 (17)	0.131522 (15)	0.03860 (7)	
Ni	0.42318 (3)	0.25520 (3)	0.37608 (3)	0.02275 (10)	
N31	0.4175 (2)	0.20616 (19)	0.25125 (19)	0.0332 (7)	
H31A	0.3959	0.2561	0.2174	0.040*	
H31B	0.4746	0.1891	0.2331	0.040*	
N32	0.3624 (2)	0.11503 (19)	0.39826 (18)	0.0293 (7)	
H32A	0.3902	0.0842	0.4428	0.035*	
H32B	0.3019	0.1228	0.4106	0.035*	
C31	0.3571 (3)	0.1199 (3)	0.2463 (2)	0.0391 (9)	
H31C	0.3696	0.0821	0.1944	0.047*	
H31D	0.2931	0.1419	0.2450	0.047*	
C32	0.3731 (3)	0.0550 (2)	0.3218 (2)	0.0358 (9)	
H32C	0.3291	−0.0005	0.3222	0.043*	
H32D	0.4350	0.0267	0.3196	0.043*	
N11A	0.5571 (3)	0.2044 (3)	0.4045 (3)	0.0245 (10)	0.707 (4)
H11A	0.5682	0.2102	0.4611	0.029*	0.707 (4)
H11B	0.5634	0.1389	0.3896	0.029*	0.707 (4)
C11A	0.6215 (5)	0.2661 (5)	0.3569 (5)	0.0366 (12)	0.707 (4)
H11C	0.6215	0.2466	0.2972	0.044*	0.707 (4)
H11D	0.6836	0.2579	0.3793	0.044*	0.707 (4)
C12A	0.5913 (4)	0.3713 (4)	0.3662 (4)	0.0398 (13)	0.707 (4)
H12A	0.5980	0.3921	0.4254	0.048*	0.707 (4)
H12B	0.6302	0.4147	0.3314	0.048*	0.707 (4)
N12A	0.4962 (4)	0.3832 (5)	0.3404 (6)	0.0336 (8)	0.707 (4)
H12C	0.4930	0.3916	0.2832	0.040*	0.707 (4)
H12D	0.4716	0.4383	0.3656	0.040*	0.707 (4)
N11B	0.5522 (9)	0.1873 (8)	0.3751 (7)	0.0245 (10)	0.293 (4)
H11E	0.5596	0.1490	0.4223	0.029*	0.293 (4)
H11F	0.5580	0.1471	0.3289	0.029*	0.293 (4)
C11B	0.6210 (11)	0.2659 (11)	0.3730 (12)	0.0366 (12)	0.293 (4)
H11G	0.6800	0.2376	0.3555	0.044*	0.293 (4)
H11H	0.6283	0.2945	0.4297	0.044*	0.293 (4)
C12B	0.5934 (8)	0.3463 (9)	0.3126 (9)	0.0398 (13)	0.293 (4)
H12E	0.6378	0.4013	0.3144	0.048*	0.293 (4)
H12F	0.5919	0.3197	0.2548	0.048*	0.293 (4)
N12B	0.5030 (10)	0.3828 (13)	0.3364 (15)	0.0336 (8)	0.293 (4)
H12G	0.4757	0.4135	0.2915	0.040*	0.293 (4)
H12H	0.5077	0.4279	0.3794	0.040*	0.293 (4)
N21A	0.4089 (4)	0.2980 (6)	0.5029 (5)	0.0373 (9)	0.707 (4)
H21A	0.3906	0.2446	0.5347	0.045*	0.707 (4)
H21B	0.4635	0.3202	0.5235	0.045*	0.707 (4)
C21A	0.3404 (4)	0.3778 (4)	0.5075 (4)	0.0418 (13)	0.707 (4)
H21C	0.3179	0.3841	0.5657	0.050*	0.707 (4)
H21D	0.3683	0.4417	0.4912	0.050*	0.707 (4)
C22A	0.2648 (4)	0.3553 (4)	0.4514 (3)	0.0352 (13)	0.707 (4)
H22A	0.2226	0.4123	0.4493	0.042*	0.707 (4)
H22B	0.2311	0.2969	0.4725	0.042*	0.707 (4)
N22A	0.3007 (5)	0.3345 (5)	0.3666 (3)	0.0279 (10)	0.707 (4)
H22C	0.3105	0.3931	0.3385	0.033*	0.707 (4)
H22D	0.2591	0.2979	0.3367	0.033*	0.707 (4)
N21B	0.3959 (10)	0.3014 (15)	0.5003 (12)	0.0373 (9)	0.293 (4)
H21E	0.4126	0.2528	0.5376	0.045*	0.293 (4)
H21F	0.4281	0.3581	0.5125	0.045*	0.293 (4)
C21B	0.2973 (9)	0.3207 (9)	0.5064 (8)	0.0418 (13)	0.293 (4)
H21G	0.2635	0.2573	0.5066	0.050*	0.293 (4)
H21H	0.2835	0.3566	0.5590	0.050*	0.293 (4)
C22B	0.2706 (12)	0.3802 (11)	0.4348 (8)	0.0352 (13)	0.293 (4)
H22E	0.3050	0.4431	0.4343	0.042*	0.293 (4)
H22F	0.2052	0.3961	0.4382	0.042*	0.293 (4)
N22B	0.2895 (13)	0.3231 (14)	0.3576 (9)	0.0279 (10)	0.293 (4)
H22G	0.2901	0.3644	0.3117	0.033*	0.293 (4)
H22H	0.2461	0.2752	0.3495	0.033*	0.293 (4)

### Atomic displacement parameters (Å^2^)

**Table:**

	*U*^11^	*U*^22^	*U*^33^	*U*^12^	*U*^13^	*U*^23^
I1	0.02763 (13)	0.04501 (14)	0.04135 (15)	−0.00301 (10)	0.00464 (11)	−0.00509 (12)
I2	0.05250 (17)	0.02837 (11)	0.03493 (14)	−0.00384 (10)	−0.00855 (12)	−0.00025 (11)
Ni	0.0228 (2)	0.01934 (17)	0.0261 (2)	0.00048 (15)	−0.00030 (19)	0.00281 (17)
N31	0.0388 (18)	0.0298 (14)	0.0311 (16)	0.0024 (13)	0.0089 (14)	−0.0018 (14)
N32	0.0307 (16)	0.0246 (13)	0.0327 (16)	−0.0070 (12)	−0.0054 (13)	0.0054 (13)
C31	0.047 (2)	0.045 (2)	0.0248 (18)	−0.0084 (18)	0.0037 (18)	−0.0089 (18)
C32	0.051 (2)	0.0238 (16)	0.033 (2)	−0.0079 (16)	0.0012 (18)	−0.0016 (16)
N11A	0.0270 (18)	0.0242 (19)	0.022 (3)	−0.0039 (15)	−0.007 (2)	−0.0052 (18)
C11A	0.035 (2)	0.0383 (17)	0.036 (3)	0.0028 (14)	0.0021 (18)	−0.0046 (18)
C12A	0.031 (3)	0.051 (3)	0.037 (3)	−0.020 (2)	0.010 (3)	0.001 (3)
N12A	0.0333 (19)	0.0277 (15)	0.0398 (19)	−0.0044 (13)	0.0086 (16)	0.0071 (15)
N11B	0.0270 (18)	0.0242 (19)	0.022 (3)	−0.0039 (15)	−0.007 (2)	−0.0052 (18)
C11B	0.035 (2)	0.0383 (17)	0.036 (3)	0.0028 (14)	0.0021 (18)	−0.0046 (18)
C12B	0.031 (3)	0.051 (3)	0.037 (3)	−0.020 (2)	0.010 (3)	0.001 (3)
N12B	0.0333 (19)	0.0277 (15)	0.0398 (19)	−0.0044 (13)	0.0086 (16)	0.0071 (15)
N21A	0.052 (2)	0.0343 (17)	0.0254 (17)	−0.0082 (18)	−0.0119 (18)	0.0056 (15)
C21A	0.055 (4)	0.033 (3)	0.037 (3)	−0.003 (2)	−0.001 (3)	−0.002 (3)
C22A	0.039 (2)	0.036 (3)	0.031 (2)	0.0025 (19)	0.0101 (19)	−0.003 (2)
N22A	0.027 (2)	0.0278 (19)	0.0286 (19)	0.0027 (16)	−0.0043 (16)	0.0020 (16)
N21B	0.052 (2)	0.0343 (17)	0.0254 (17)	−0.0082 (18)	−0.0119 (18)	0.0056 (15)
C21B	0.055 (4)	0.033 (3)	0.037 (3)	−0.003 (2)	−0.001 (3)	−0.002 (3)
C22B	0.039 (2)	0.036 (3)	0.031 (2)	0.0025 (19)	0.0101 (19)	−0.003 (2)
N22B	0.027 (2)	0.0278 (19)	0.0286 (19)	0.0027 (16)	−0.0043 (16)	0.0020 (16)

### Geometric parameters (Å, °)

**Table:**

Ni—N31	2.101 (3)	N11B—H11E	0.9200
Ni—N22A	2.105 (7)	N11B—H11F	0.9200
Ni—N11B	2.113 (13)	C11B—C12B	1.507 (14)
Ni—N12A	2.113 (7)	C11B—H11G	0.9900
Ni—N21A	2.116 (7)	C11B—H11H	0.9900
Ni—N21B	2.117 (19)	C12B—N12B	1.472 (14)
Ni—N32	2.122 (3)	C12B—H12E	0.9900
Ni—N11A	2.140 (5)	C12B—H12F	0.9900
Ni—N12B	2.180 (18)	N12B—H12G	0.9200
Ni—N22B	2.194 (18)	N12B—H12H	0.9200
N31—C31	1.467 (4)	N21A—C21A	1.478 (8)
N31—H31A	0.9200	N21A—H21A	0.9200
N31—H31B	0.9200	N21A—H21B	0.9200
N32—C32	1.473 (4)	C21A—C22A	1.462 (8)
N32—H32A	0.9200	C21A—H21C	0.9900
N32—H32B	0.9200	C21A—H21D	0.9900
C31—C32	1.508 (5)	C22A—N22A	1.482 (6)
C31—H31C	0.9900	C22A—H22A	0.9900
C31—H31D	0.9900	C22A—H22B	0.9900
C32—H32C	0.9900	N22A—H22C	0.9200
C32—H32D	0.9900	N22A—H22D	0.9200
N11A—C11A	1.473 (7)	N21B—C21B	1.481 (14)
N11A—H11A	0.9200	N21B—H21E	0.9200
N11A—H11B	0.9200	N21B—H21F	0.9200
C11A—C12A	1.495 (8)	C21B—C22B	1.450 (14)
C11A—H11C	0.9900	C21B—H21G	0.9900
C11A—H11D	0.9900	C21B—H21H	0.9900
C12A—N12A	1.470 (8)	C22B—N22B	1.480 (14)
C12A—H12A	0.9900	C22B—H22E	0.9900
C12A—H12B	0.9900	C22B—H22F	0.9900
N12A—H12C	0.9200	N22B—H22G	0.9200
N12A—H12D	0.9200	N22B—H22H	0.9200
N11B—C11B	1.467 (14)		
			
N31—Ni—N22A	93.25 (16)	C11A—C12A—H12A	109.4
N31—Ni—N11B	83.9 (3)	N12A—C12A—H12B	109.4
N22A—Ni—N11B	173.3 (4)	C11A—C12A—H12B	109.4
N31—Ni—N12A	91.3 (3)	H12A—C12A—H12B	108.0
N22A—Ni—N12A	90.2 (2)	C12A—N12A—Ni	108.7 (4)
N11B—Ni—N12A	83.8 (3)	C12A—N12A—H12C	109.9
N31—Ni—N21A	171.6 (2)	Ni—N12A—H12C	109.9
N22A—Ni—N21A	81.1 (2)	C12A—N12A—H12D	109.9
N11B—Ni—N21A	102.4 (4)	Ni—N12A—H12D	109.9
N12A—Ni—N21A	94.9 (3)	H12C—N12A—H12D	108.3
N31—Ni—N21B	166.7 (4)	C11B—N11B—Ni	108.0 (9)
N22A—Ni—N21B	75.8 (4)	C11B—N11B—H11E	110.1
N11B—Ni—N21B	107.8 (5)	Ni—N11B—H11E	110.1
N12A—Ni—N21B	96.2 (6)	C11B—N11B—H11F	110.1
N21A—Ni—N21B	5.4 (5)	Ni—N11B—H11F	110.1
N31—Ni—N32	81.99 (11)	H11E—N11B—H11F	108.4
N22A—Ni—N32	95.9 (2)	N11B—C11B—C12B	110.4 (12)
N11B—Ni—N32	89.7 (3)	N11B—C11B—H11G	109.6
N12A—Ni—N32	171.2 (2)	C12B—C11B—H11G	109.6
N21A—Ni—N32	92.4 (2)	N11B—C11B—H11H	109.6
N21B—Ni—N32	91.5 (6)	C12B—C11B—H11H	109.6
N31—Ni—N11A	97.93 (14)	H11G—C11B—H11H	108.1
N22A—Ni—N11A	166.02 (19)	N12B—C12B—C11B	108.7 (12)
N11B—Ni—N11A	14.3 (3)	N12B—C12B—H12E	110.0
N12A—Ni—N11A	81.26 (19)	C11B—C12B—H12E	110.0
N21A—Ni—N11A	88.6 (2)	N12B—C12B—H12F	110.0
N21B—Ni—N11A	94.0 (4)	C11B—C12B—H12F	110.0
N32—Ni—N11A	93.98 (14)	H12E—C12B—H12F	108.3
N31—Ni—N12B	89.7 (6)	C12B—N12B—Ni	107.5 (10)
N22A—Ni—N12B	92.4 (4)	C12B—N12B—H12G	110.2
N11B—Ni—N12B	81.6 (4)	Ni—N12B—H12G	110.2
N12A—Ni—N12B	2.6 (6)	C12B—N12B—H12H	110.2
N21A—Ni—N12B	96.7 (7)	Ni—N12B—H12H	110.2
N21B—Ni—N12B	98.2 (8)	H12G—N12B—H12H	108.5
N32—Ni—N12B	168.6 (5)	C21A—N21A—Ni	108.3 (4)
N11A—Ni—N12B	79.4 (4)	C21A—N21A—H21A	110.0
N31—Ni—N22B	88.1 (4)	Ni—N21A—H21A	110.0
N22A—Ni—N22B	6.7 (5)	C21A—N21A—H21B	110.0
N11B—Ni—N22B	171.8 (5)	Ni—N21A—H21B	110.0
N12A—Ni—N22B	94.6 (5)	H21A—N21A—H21B	108.4
N21A—Ni—N22B	85.7 (4)	C22A—C21A—N21A	109.8 (5)
N21B—Ni—N22B	80.4 (5)	C22A—C21A—H21C	109.7
N32—Ni—N22B	90.9 (5)	N21A—C21A—H21C	109.7
N11A—Ni—N22B	172.7 (4)	C22A—C21A—H21D	109.7
N12B—Ni—N22B	96.7 (6)	N21A—C21A—H21D	109.7
C31—N31—Ni	109.0 (2)	H21C—C21A—H21D	108.2
C31—N31—H31A	109.9	C21A—C22A—N22A	109.1 (5)
Ni—N31—H31A	109.9	C21A—C22A—H22A	109.9
C31—N31—H31B	109.9	N22A—C22A—H22A	109.9
Ni—N31—H31B	109.9	C21A—C22A—H22B	109.9
H31A—N31—H31B	108.3	N22A—C22A—H22B	109.9
C32—N32—Ni	107.9 (2)	H22A—C22A—H22B	108.3
C32—N32—H32A	110.1	C22A—N22A—Ni	109.7 (4)
Ni—N32—H32A	110.1	C22A—N22A—H22C	109.7
C32—N32—H32B	110.1	Ni—N22A—H22C	109.7
Ni—N32—H32B	110.1	C22A—N22A—H22D	109.7
H32A—N32—H32B	108.4	Ni—N22A—H22D	109.7
N31—C31—C32	108.8 (3)	H22C—N22A—H22D	108.2
N31—C31—H31C	109.9	C21B—N21B—Ni	107.4 (10)
C32—C31—H31C	109.9	C21B—N21B—H21E	110.2
N31—C31—H31D	109.9	Ni—N21B—H21E	110.2
C32—C31—H31D	109.9	C21B—N21B—H21F	110.2
H31C—C31—H31D	108.3	Ni—N21B—H21F	110.2
N32—C32—C31	109.0 (3)	H21E—N21B—H21F	108.5
N32—C32—H32C	109.9	C22B—C21B—N21B	108.2 (12)
C31—C32—H32C	109.9	C22B—C21B—H21G	110.1
N32—C32—H32D	109.9	N21B—C21B—H21G	110.1
C31—C32—H32D	109.9	C22B—C21B—H21H	110.1
H32C—C32—H32D	108.3	N21B—C21B—H21H	110.1
C11A—N11A—Ni	107.8 (3)	H21G—C21B—H21H	108.4
C11A—N11A—H11A	110.2	C21B—C22B—N22B	108.5 (12)
Ni—N11A—H11A	110.2	C21B—C22B—H22E	110.0
C11A—N11A—H11B	110.2	N22B—C22B—H22E	110.0
Ni—N11A—H11B	110.2	C21B—C22B—H22F	110.0
H11A—N11A—H11B	108.5	N22B—C22B—H22F	110.0
N11A—C11A—C12A	107.0 (5)	H22E—C22B—H22F	108.4
N11A—C11A—H11C	110.3	C22B—N22B—Ni	105.9 (10)
C12A—C11A—H11C	110.3	C22B—N22B—H22G	110.5
N11A—C11A—H11D	110.3	Ni—N22B—H22G	110.5
C12A—C11A—H11D	110.3	C22B—N22B—H22H	110.5
H11C—C11A—H11D	108.6	Ni—N22B—H22H	110.5
N12A—C12A—C11A	111.1 (5)	H22G—N22B—H22H	108.7
N12A—C12A—H12A	109.4		
			
N22A—Ni—N31—C31	−81.5 (3)	C11B—C12B—N12B—Ni	−38.7 (17)
N11B—Ni—N31—C31	104.6 (4)	N31—Ni—N12B—C12B	−70.9 (13)
N12A—Ni—N31—C31	−171.7 (3)	N22A—Ni—N12B—C12B	−164.2 (13)
N21A—Ni—N31—C31	−34.1 (14)	N11B—Ni—N12B—C12B	12.9 (13)
N21B—Ni—N31—C31	−47 (2)	N12A—Ni—N12B—C12B	162 (18)
N32—Ni—N31—C31	14.0 (2)	N21A—Ni—N12B—C12B	114.5 (13)
N11A—Ni—N31—C31	106.9 (3)	N21B—Ni—N12B—C12B	119.8 (13)
N12B—Ni—N31—C31	−173.8 (4)	N32—Ni—N12B—C12B	−28 (4)
N22B—Ni—N31—C31	−77.1 (6)	N11A—Ni—N12B—C12B	27.2 (12)
N31—Ni—N32—C32	14.7 (2)	N22B—Ni—N12B—C12B	−159.0 (13)
N22A—Ni—N32—C32	107.2 (3)	N31—Ni—N21A—C21A	−63.5 (16)
N11B—Ni—N32—C32	−69.1 (4)	N22A—Ni—N21A—C21A	−15.4 (5)
N12A—Ni—N32—C32	−25.7 (15)	N11B—Ni—N21A—C21A	158.8 (5)
N21A—Ni—N32—C32	−171.5 (3)	N12A—Ni—N21A—C21A	74.0 (5)
N21B—Ni—N32—C32	−176.9 (4)	N21B—Ni—N21A—C21A	−30 (8)
N11A—Ni—N32—C32	−82.7 (3)	N32—Ni—N21A—C21A	−111.0 (5)
N12B—Ni—N32—C32	−29 (3)	N11A—Ni—N21A—C21A	155.1 (5)
N22B—Ni—N32—C32	102.7 (4)	N12B—Ni—N21A—C21A	75.9 (6)
Ni—N31—C31—C32	−39.9 (3)	N22B—Ni—N21A—C21A	−20.3 (7)
Ni—N32—C32—C31	−40.5 (3)	Ni—N21A—C21A—C22A	41.1 (6)
N31—C31—C32—N32	54.4 (4)	N21A—C21A—C22A—N22A	−52.7 (7)
N31—Ni—N11A—C11A	69.7 (4)	C21A—C22A—N22A—Ni	37.8 (6)
N22A—Ni—N11A—C11A	−73.1 (10)	N31—Ni—N22A—C22A	161.9 (4)
N11B—Ni—N11A—C11A	79.1 (15)	N11B—Ni—N22A—C22A	−134 (3)
N12A—Ni—N11A—C11A	−20.4 (4)	N12A—Ni—N22A—C22A	−106.9 (5)
N21A—Ni—N11A—C11A	−115.6 (4)	N21A—Ni—N22A—C22A	−11.9 (5)
N21B—Ni—N11A—C11A	−116.1 (7)	N21B—Ni—N22A—C22A	−10.5 (7)
N32—Ni—N11A—C11A	152.1 (4)	N32—Ni—N22A—C22A	79.6 (4)
N12B—Ni—N11A—C11A	−18.5 (7)	N11A—Ni—N22A—C22A	−55.0 (11)
N22B—Ni—N11A—C11A	−76 (4)	N12B—Ni—N22A—C22A	−108.3 (8)
Ni—N11A—C11A—C12A	44.7 (6)	N22B—Ni—N22A—C22A	121 (5)
N11A—C11A—C12A—N12A	−54.8 (7)	N31—Ni—N21B—C21B	−13 (3)
C11A—C12A—N12A—Ni	36.2 (7)	N22A—Ni—N21B—C21B	22.5 (10)
N31—Ni—N12A—C12A	−106.1 (5)	N11B—Ni—N21B—C21B	−163.3 (10)
N22A—Ni—N12A—C12A	160.6 (5)	N12A—Ni—N21B—C21B	111.1 (11)
N11B—Ni—N12A—C12A	−22.4 (6)	N21A—Ni—N21B—C21B	−172 (9)
N21A—Ni—N12A—C12A	79.5 (5)	N32—Ni—N21B—C21B	−73.1 (12)
N21B—Ni—N12A—C12A	84.8 (6)	N11A—Ni—N21B—C21B	−167.2 (11)
N32—Ni—N12A—C12A	−66.1 (18)	N12B—Ni—N21B—C21B	112.9 (12)
N11A—Ni—N12A—C12A	−8.3 (5)	N22B—Ni—N21B—C21B	17.5 (12)
N12B—Ni—N12A—C12A	−53 (17)	Ni—N21B—C21B—C22B	−47.1 (15)
N22B—Ni—N12A—C12A	165.6 (6)	N21B—C21B—C22B—N22B	61.4 (18)
N31—Ni—N11B—C11B	106.4 (10)	C21B—C22B—N22B—Ni	−42.9 (16)
N22A—Ni—N11B—C11B	41 (3)	N31—Ni—N22B—C22B	−173.6 (11)
N12A—Ni—N11B—C11B	14.4 (10)	N22A—Ni—N22B—C22B	−34 (4)
N21A—Ni—N11B—C11B	−79.3 (11)	N11B—Ni—N22B—C22B	−161 (3)
N21B—Ni—N11B—C11B	−80.2 (12)	N12A—Ni—N22B—C22B	−82.5 (12)
N32—Ni—N11B—C11B	−171.7 (10)	N21A—Ni—N22B—C22B	12.2 (11)
N11A—Ni—N11B—C11B	−64.3 (15)	N21B—Ni—N22B—C22B	13.1 (12)
N12B—Ni—N11B—C11B	15.8 (12)	N32—Ni—N22B—C22B	104.5 (11)
N22B—Ni—N11B—C11B	94 (4)	N11A—Ni—N22B—C22B	−27 (5)
Ni—N11B—C11B—C12B	−42.4 (16)	N12B—Ni—N22B—C22B	−84.1 (13)
N11B—C11B—C12B—N12B	55.6 (19)		

### Hydrogen-bond geometry (Å, °)

**Table:**

*D*—H···*A*	*D*—H	H···*A*	*D*···*A*	*D*—H···*A*
N31—H31A···I2	0.92	2.83	3.731 (3)	167
N31—H31B···I1	0.92	2.79	3.663 (3)	158
N32—H32A···I2^i^	0.92	3.05	3.786 (3)	138
N32—H32B···I1^ii^	0.92	2.86	3.724 (3)	157
N11A—H11B···I2^iii^	0.92	2.97	3.854 (4)	162
N12A—H12C···I2	0.92	3.15	3.940 (8)	145
N11B—H11F···I2^iii^	0.92	3.15	3.619 (12)	114
N12B—H12G···I2	0.92	3.07	3.94 (2)	159
N12B—H12H···I1^iv^	0.92	3.27	3.824 (16)	121
N21A—H21A···I2^i^	0.92	2.92	3.826 (8)	171
N21A—H21B···I1^i^	0.92	2.77	3.665 (7)	166
N22A—H22D···I1^ii^	0.92	3.19	3.905 (7)	136
N21B—H21E···I2^i^	0.92	3.05	3.86 (2)	148
N21B—H21F···I1^i^	0.92	3.22	3.832 (16)	125
N22B—H22G···I2	0.92	3.21	4.061 (16)	154
N22B—H22H···I1^ii^	0.92	2.80	3.69 (2)	163

Symmetry codes: (i) *x*, −*y*+1/2, *z*+1/2; (ii) *x*−1/2, *y*, −*z*+1/2; (iii) −*x*+1, *y*−1/2, −*z*+1/2; (iv) −*x*+1, *y*+1/2, −*z*+1/2.
